# What medicines do households keep in their cabinets? Understanding the possession and use of medicines at home and the role of health insurance in Nigeria

**DOI:** 10.1371/journal.pone.0247591

**Published:** 2021-02-24

**Authors:** Hezekiah Olayinka Shobiye, Oladimeji Akeem Bolarinwa, Mojirola Martina Fasiku, Tanimola Makanjuola Akande, Wendy Janssens

**Affiliations:** 1 John F. Kennedy School of Government, Harvard University, Boston, Massachusetts, United States of America; 2 Department of Global Health, Boston University School of Public Health, Boston, Massachusetts, United States of America; 3 Department of Epidemiology and Community Health, University of Ilorin Teaching Hospital, Ilorin, Nigeria; 4 Department of Community Medicine and Primary Care, Federal Medical Centre, Abeokuta, Ogun, Nigeria; 5 Amsterdam Institute for Global Health and Development (AIGHD), Amsterdam, The Netherlands; 6 School of Business and Economics, Vrije Universiteit Amsterdam, The Netherlands; University of West London, UNITED KINGDOM

## Abstract

**Background:**

Globally, the possession of medicines stored at home is increasing. However, little is known about the determinants of possessing medicines, their usage according to clinical purpose, which we term ‘correct drug match’, and the role of health insurance.

**Methods:**

This study uses data from a 2013 survey evaluating a health insurance program in Kwara State, Nigeria, which upgraded health facilities and subsidized insurance premiums. The final dataset includes 1,090 households and 4,641 individuals. Multilevel mixed-effects logistic regressions were conducted at both the individual level and at the level of the medicines kept in respondents’ homes to understand the determinants of medicine possession and correct drug match, respectively, and to investigate the effect of health insurance on both.

**Results:**

A total of 9,266 medicines were classified with 61.2% correct match according to self-reported use, 11.9% incorrect match and 26.9% indeterminate. Most medicines (73.0%) were obtained from patent proprietary medicine vendors (PPMVs). At 36.6%, analgesics were the most common medicine held at home, while anti-malarial use had the highest correct match at 96.1%. Antihistamines, vitamins and minerals, expectorants, and antibiotics were most likely to have an incorrect match at respectively 35.8%, 33.6%, 31.9%, and 26.6%. Medicines were less likely to have a correct match when found with the uneducated and obtained from public facilities. Enrolment in the insurance program increased correct matches for specific medicines, notably antihypertensives and antibiotics (odds ratio: 25.15 and 3.60, respectively).

**Conclusion:**

Since PPMVs serve as both the most popular and better channel compared to the public sector to obtain medicines, we recommend that policymakers strengthen their focus on these vendors to educate communities on medicine types and their correct use. Health insurance programs that provide affordable access to improved-quality health facilities represent another important avenue for reducing the burden of incorrect drug use. This appears increasingly important in view of the global rise in antimicrobial resistance.

## Introduction

Over the years, there has been an increasing global trend in the number of households with medicines at home [1–4]. This has been attributed to increased accessibility, especially from informal channels like proprietary patent medicine vendors (PPMVs) and alternative healthcare providers, which are common in developing countries [5–8]. Unlike the medicines obtained from the formal sector such as hospitals and clinics, and from registered pharmacies, those obtained in the informal sector are largely characterized by their inconsistent quality and high risk of inappropriate use [9–11]. This is because this sector is loosely regulated, mostly profit-driven, and often lacks the presence of a qualified medical practitioner to provide proper guidance and diagnosis for the use of the medicines [12, 13]. For medicines prescribed through the formal sector there are other risks, like over-prescription, based on presumptive treatment decisions in the absence of proper diagnostic tests, leading not only to unnecessary expenditures and potential drug resistance, but also to increased amounts of medicines kept at home [14, 15].

Having medicines at home is linked with irrational use and misuse [1, 16, 17], which increases the risk of adverse effects [18, 19], especially when patients consume medicines without an adequate understanding of the active ingredients and clinical purpose [16]. In turn this may increase the burden on the health system and antimicrobial resistance [19–21].

Existing studies show that households keep different types of medicines at home, with analgesics being the most commonly found at home [22]. Other commonly used medicines were antibiotics [23], and vitamins, minerals and herbal preparations [22]. None of these studies examined whether the medicines kept at home were used according to their clinical purpose.

Sociodemographic factors and morbidity status influence the possession and use of medicines at home, such as being of older age [24] or suffering from a chronic disease [22]. Evidence with respect to the association between gender and self-medication is inconclusive [22, 25]. Households with higher education had the tendency to obtain medicines from the hospitals and pharmacies, while those with lower education levels had the tendency to obtain medicines from PPMVs and local hawkers [26]. Married individuals were more likely than the unmarried to obtain their medicines from PPMVs rather than from hospitals or pharmacies [26].

Impact evaluations of health insurance programs have shown that insurance status is associated with increased formal healthcare utilization [27, 28], which may suggest reduced misuse of medicines because of increased access to better advice from qualified medical professionals. However, studies have also shown that increasing access to formal healthcare through health insurance does not reduce the consumption of informal care from self-medication and treatment within the household [8, 29]. In fact, having health insurance may also increase the likelihood of possessing left-over medicines since it can improve access to formal healthcare and affordability of treatments [30]. Despite some evidence on the association between insurance status and the presence of medicines at home, it remains unclear whether these medicines are actually used correctly for the intended purpose. Understanding the role of health insurance in appropriate medicine use is thus important, particularly in the context of universal health coverage (UHC) since health insurance is increasingly promoted as an important risk-pooling mechanism.

Finding answers to the burden of irrational use of medicines in Nigeria and other low- and middle-income countries is particularly important as many decision-makers are unaware of the magnitude, and the economic and health costs associated with it [9]. For this paper, we narrowly define a drug match to be correct when the reported use of a medicine aligns with the clinical description of conditions for which the medicine can be used, and incorrect to be otherwise. To that end, this study aims to understand the factors that influence having medicines at home, the extent of a correct match and the factors that determine this match. In addition, this study examines whether the likelihood of having medicines at home or having a correct match is different for medicines used by individuals with or without health insurance.

We used a unique dataset collected in Nigeria in 2013 called “Medication Cabinet” data to generate evidence that can be used by policymakers in Nigeria and elsewhere in the region to address the menace of irrational drug use. These data are particularly suited for our purposes because they were collected within the context of an impact evaluation of a health insurance program that both subsidized the insurance premium for households in program areas and upgraded the quality of selected facilities. As such, the data explicitly allow for an assessment of the role of insurance on medicine use at home.

## Research design and methodology

### Study setting and design

The Medication Cabinet data was collected as part of a large and representative household survey administered in 2013 in Kwara Central senatorial zone in Nigeria to evaluate the endline impact of the Kwara State Health Insurance Plan (KSHIP). This subsidized community-based health insurance program was funded by the Dutch Health Insurance Fund (HIF) and implemented in collaboration with the Kwara State Government, PharmAccess Foundation and the Nigerian Health Maintenance Organization (HMO), Hygeia Ltd. The program was unique in that it consisted of insurance subsidies (i.e. a demand-side intervention) and simultaneous quality upgrades of selected clinics (i.e. a supply-side intervention) [31]. Both types of interventions might impact the amount of medicines at home as well as correct drug match, albeit through different channels–being reduced price of consultations and medication, and improved quality of care.

The study was conducted in the two districts of Afon and Aboto Oja where KSHIP was rolled out since 2009 (treatment areas), as well as in the district of Ajasse Ipo where KSHIP was not rolled out yet (the control area). Ajasse Ipo district was selected as a comparison district after a scan conducted by the research team at baseline. This district was most similar to the treatment areas in terms of language, socioeconomic characteristics, distribution of clinics, urban/rural composition and population size. Several studies have evaluated the impact of the insurance program on outcomes such as health care utilization and out-of-pocket spending [27], catastrophic health expenditures [32], and hypertension [33].

### Sampling methodology and sample size

The study sample was set up as a representative self-weighted sample of the target population in the three districts, based on a stratified two-stage clustered random sampling methodology at the baseline survey in 2009. First, in each district a complete list of enumeration areas (EAs) was obtained from the 2005 Nigerian National Population Census. Only EAs within a 15 km radius of the main health facility in the district were kept in the sampling frame. From these sampling frames, 30 EAs were randomly selected in each of the two treatment districts, and 40 EAs were randomly selected in the comparison district–yielding a total number of 100 EAs.

Next, a household listing exercise was conducted in all selected EAs. From these listings, the research team randomly sampled 15 households per EA on average. The precise number of households sampled within an EA was proportional to the EA’s relative population size, such that more households were sampled from larger EAs even though all households in the study area had the same probability of being included in the study. This resulted in a total sample size at baseline of 900 households in the treatment districts and 600 households in the comparison district (due to the oversampling of treatment EAs).

### Data collection and field work

Surveys were conducted in the treatment and control areas in 2009, 2011 and 2013. The 2013 questionnaire contained a module for the Medication Cabinet study. The interviewers went into the field in teams. Each team consisted of one supervisor and three interviewer pairs of a biomedical and socioeconomic enumerator. The supervisors monitored the fieldwork and performance of enumerators. The household members were interviewed in private to make respondents comfortable and have the privacy to respond to the questions. Supervisors observed household interviews, inspected completed questionnaires, and provided additional training to interviewers if needed.

All medicines present in the household were recorded and for each medicine several questions were recorded: the individual user of the medicine, the name of medicine, where the medicine was kept, the health condition it was used for and lastly, where it was obtained. The interviewers identified the medicines using one or more of 4 methods. First, the respondents were asked for the name of the medicine; second, the name on the pack was examined; third, the prescription received from a healthcare provider if any was looked at and fourth, through a sample medicine. As much as possible, interviewers used a combination of these methods.

The Medication Cabinet data was matched to the individual and household demographic, socio-economic and health data from the household survey. In the final endline dataset used for the Medication Cabinet study, there were 4,641 individuals and 1,090 distinct households representing an attrition rate of 27.3% from the baseline sample size of 1,500 households. All data were deidentified before accessing them for analysis.

Ethical approval for the study was obtained from the Ethical and Research Committee (ERC) of the University of Ilorin Teaching Hospital (UITH), Nigeria (UITH/CAT/189/11/782). Written informed consent was obtained from adult household members before households were included in the survey. For those under 18, consent was obtained from the head of household.

### Data management and analysis

To answer our research questions, we conducted both descriptive and multiple regression analyses. The descriptive analyses documented the most common medicines used at home and whether the use of these medicines matched the range of clinical use. For the regression analyses, we used multilevel mixed-effects models to answer two main questions, further specified below. Multilevel mixed-effects models were used to control for the common cluster-level random effects among observations since individuals were clustered within households and individual medicines were clustered within an individual.

For the first research question, we wanted to understand the predictors of **having medicines at home** at the individual level. For this, the dependent variable was having medicine at home or not, and the unit of observation was the individual. The independent variables included demographic, socioeconomic and health-related factors such as age group (categorical variables for 0–18, 19–35, 36–60 and older than 60), gender, being married, household size (binary variable equal to 1 for household sizes greater than the Nigerian average of 5 members [34]), highest completed education level (no education or primary incomplete; primary; secondary; tertiary or higher), income level (assessed by measuring per capita annual consumption, and assigning individuals into five quintiles from poorest (1) to richest (5)), morbidity status (binary variable equal to 1 if suffering from at least one chronic disease and 0 otherwise), and the distance to the nearest health facility (in km).

To assess the association with health insurance, the regressions also included indicators of whether the individual lived in the treatment area or in the control area, and whether the individual was enrolled in health insurance at the time of the interview or not. Virtually no one in the control area (n = 2) was insured at the time of the endline survey. Hence, individuals with health insurance (benefitting from the financial coverage of the insurance scheme) were considered as a subset of all individuals living in the treatment area (benefitting from the quality upgrades in their nearby facility, irrespective of insurance status). Enrolment in health insurance was a voluntary decision, hence regression coefficients are considered to reflect association rather than causation. To understand whether the main effects seen were reinforced or instead attenuated by the health insurance scheme, we ran additional specifications in which the program variables (i.e. living in the treatment area and having health insurance) were interacted with each of the other predictor variables.

The second multilevel mixed-effects logistic regression model was conducted to provide insights into the determinants of a **correct drug match** at the level of the medicines kept in respondents’ homes. As shown in Table 1, a drug match was determined to be correct when the respondent’s reported use of the medicine corresponds with the clinical description of conditions for which the medicine can be used (i.e. when the reported use was indicated on the medication; or when the medicine was used for symptoms that can arise because of the condition, or for another medical condition that can be caused by the index medical condition). In this definition, we did not claim that a correct match indicates that the medicine was correctly used based on actual diagnosis, because most respondents did not receive an actual diagnosis. A medicine was coded as indeterminable when the reported use could not be verified through the medical practice in the study site; or when it was a herbal drug. Indeterminable medicines were not included in the second regression model.

**Table 1:** Guidelines to determine correct, incorrect and indeterminate drug match based on local epidemiological and clinical context.

Drug Class	Correct Drug Match
Analgesics	Pain, injury, Boil, Headache, Abdominal pain, Arthritis, and Postoperative use
Analgesic (Paracetamol only)	Febrile illness, Malaria (because of the anti-pyretic effect)
Antacids	Heart burns, Ulcer diseases and Pregnancy
Anti-asthmatic	Relief of asthmatic episode including breathlessness symptoms, Cough
Antidiabetics	Diabetes (sugar disease)
Antihistamines	Catarrh, Running nose, Peptic Ulcer Disease/Abdominal pain
Antacids	Ulcers, Stomachache, Pregnancy (Antacids are commonly used for pregnancy induced gastritis)
Antihypertensives	Hypertension
Antimalarial	Malaria, Febrile illness, Pregnancy (Antimalarial is routinely prescribed for pregnancy in the study setting)
Antispasmodics	Muscle pain/discomfort, Abdominal pain/discomfort, Menstrual pain
Antibiotics^a^	Febrile illness, Eye infection, Gastrointestinal infections like diarrhea and dysentery, Respiratory tract infections like catarrh, cough and pneumonia, Ulcer, Wounds/injury, Typhoid, Boil, Postoperative use, Pile (local language for various Gastrointestinal symptoms)
Expectorants	Cough
Haematinics	Blood supplements, Pregnancy, Injury/wound, Febrile illness
Vitamins/Minerals	Appetite stimulant, Healthy living, Pregnancy, Febrile illness, Injury/wound
Vitamin C	Cold, Catarrh
	**Incorrect Drug Match**
Antispasmodics	Febrile illness
Antihistamines	Febrile Illness, Sleeplessness, Pain
Analgesic	Hypertension
Haematinics	Cold, Catarrh, Pain
Vitamins/Minerals	Asthma, Diarrhea, Hypertension, Skin problem, Sleeplessness, Sugar disease, TB, Ulcer
Antihypertensive	Malaria, Febrile illness
Antibiotics	Malaria, Sugar disease, Hypertension, Skin problem, Pain
	**Indeterminate Drug Match**
Analgesic	Ulcer disease
Analgesic (Paracetamol only)	Malaria, Febrile illness
Haematinics/Vitamins/ Minerals	Malaria, Febrile illness
Antihistamine	Malaria (in the study setting, antihistamines are prescribed along-side antimalarial especially those that still use chloroquine), Pain (inconclusive with the type of pain as it is possible that it is an abdominal pain)
Antibiotics	Skin problem (Skin problem can also be a skin disease, which may require antibiotics use)
Herbal and Alternative/ Complementary drugs	Common clinical conditions and symptoms in the study setting
All medication class	If response is “Don’t know” or response space is blank

^a^In Nigeria, antibiotics are commonly prescribed for diarrhea, respiratory tract infections and febrile illnesses that are not malaria.

The classification of drug match was a complex process that took into consideration the limitations of the epidemiological and clinical context of the study area–such as the local prescription practice being characterized by polypharmacy, which is a concurrent use of multiple medications by a patient for a medical condition. Three independent clinicians (one clinical professor and two medical consultants from the study area) generated the guidelines in Table 1 and coded the responses accordingly. The unit of observation in this analysis was each of the medicines kept in respondents’ homes. The independent and predictor variables included the drug class, place of purchase, demographic, socio-economic, and health- and insurance-related variables as described in the previous paragraphs.

All analyses were done using Stata 16. Chi-square and t-test statistics were used to assess the relationship between the predictor variables and the dependent variable. The deviance of the model was used to compute the fit for the logistic regression models. The lower the deviance, the better the ability of the model to predict the observed outcome [35]. In addition, robust standard errors were used to correct for clustering at the household and individual levels, and outliers in the dataset [36].

## Results

### Description of the study population

The sample characteristics are shown in Table 2 Column (1). In the study sample, there were 4,641 individual respondents from 1,090 households. Approximately half of individuals (51%) were aged 18 and below, 43% had no education and nearly two-thirds (64%) were single. The percentage of females (52%) was slightly higher than males (48%). In addition, about half (51%) lived in a household size between 1 and 5, and the majority (90%) had no chronic disease. Nearly two-thirds of respondents (64%) lived in a treatment area and about 1 in every 5 people was enrolled in health insurance at endline. Almost everyone with health insurance (950/952 = 99.8%) lived in a treatment area.

**Table 2:** Characteristics of respondents.

	Total	Medicines at Home		
	N (%)	No n (%)	Yes n (%)	
	4,641 (100%)	1,283 (27.6%)	3,358 (72.4%)	P-value
Demographic Characteristics				
**Age Group**				
0–18	2,373 (51.1%)	791 (33.3%)	1,582 (66.7%)	< .0001
19–35	648 (14.0%)	193 (29.8%)	455 (70.2%)	
36–60	1,004 (21.6%)	197 (19.6%)	807 (80.4%)	
≥61	616 (13.3%)	102 (16.6%)	514 (83.4%)	
**Gender**				
Male	2,233 (48.1%)	647 (29.0%)	1,586 (71.0%)	0.051
Female	2,408 (51.9%)	636 (26.4%)	1,772 (73.6%)	
Socioeconomic Characteristics				
**Marital Status**				
Single	2,947 (63.6%)	936 (31.8%)	2,011 (68.2%)	< .0001
Married	1,684 (36.4%)	342 (20.3%)	1,342 (79.7%)	
Missing	10	5	5	
**Household Size**				
1–5	2,384 (51.4%)	591 (24.8%)	1,793 (75.2%)	< .0001
≥6	2,257 (48.6%)	692 (30.7%)	1,565 (69.3%)	
**Education Level**				
No education	2,003 (43.3%)	481 (24.5%)	1,482 (75.5%)	< .0001
Primary	1,397 (30.8%)	422 (30.2%)	975 (69.8%)	
Secondary	973 (21.5%)	318 (32.7%)	655 (67.3%)	
Tertiary	202 (4.5%)	31 (15.4%)	171 (84.6%)	
Missing	106	31	75	
**Income Level**				
Quintile 1 (Poorest)	428 (9.2%)	144 (33.6%)	284 (66.4%)	< .0001
Quintile 2	529 (11.4%)	145 (27.4%)	384 (72.6%)	
Quintile 3	888 (19.2%)	261 (29.4%)	627 (70.6%)	
Quintile 4	1,021 (22.0%)	309 (30.3%)	712 (69.7%)	
Quintile 5 (Richest)	1,772 (38.2%)	422 (23.8%)	1,350 (76.2%)	
Missing	3	2	1	
Health-related Characteristics				
**Chronic Disease**				
No	4,157 (89.9%)	1,207 (29.0%)	2,950 (71.0%)	< .0001
Yes, one or more	468 (10.1%)	68 (12.8%)	408 (87.2%)	
Missing	16	16	-	
**Distance to a Health Facility (Km)**				
Nearest health facility (Mean)	4,634 (1.40)	1,283 (1.42)	3,351 (1.40)	0.791
Missing	7	-	7	
Intervention Characteristics				
**Living in a Treatment Area**				
No	1,673 (36.1%)	262 (15.7%)	1,411 (84.3%)	< .0001
Yes	2,968 (63.9%)	1,021 (34.4%)	1,947 (65.6%)	
**Insurance Status**				
No	3,675 (79.4%)	1,001 (27.2%)	2,674 (72.8%)	0.449
Yes	952 (20.6%)	271 (28.5%)	681 (71.5%)	
Missing	14	11	3	
**Insurance Status** (Living in a treatment area, n = 2,957)				
No	2,007 (67.9%)	742 (37.0%)	1,265 (63.0%)	< .0001
Yes	950 (32.1%)	269 (28.3%)	681 (71.7%)	

**Fig 1:**
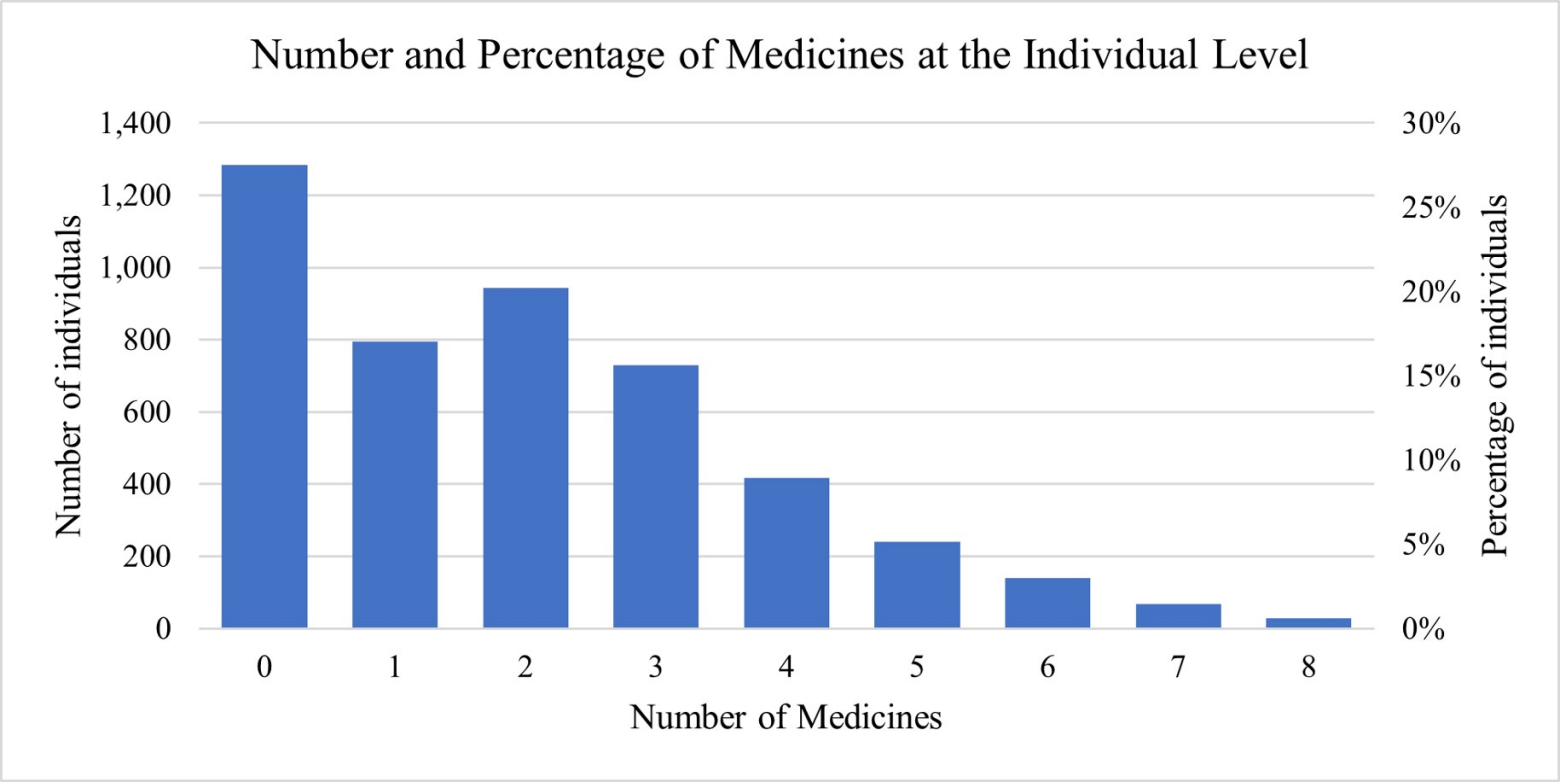
Number and percentage of medicines at the individual level.

From the total sample, 72.4% of individuals had at least one medicine at home. The mean number of medicines held at home per person was 2.0 (SD = 1.8). As shown in Fig 1, 17% of individuals had only one medicine at home, 20% had two medicines, 16% had three medicines, and 9% had four medicines. The highest number of medicines found with a person was 8.

### Characteristics of medicines found at home

In total, 9,266 medicines were held at home by the respondents. These medicines belonged to 13 classes of drugs. From Table 3 Panel A, analgesics represented the highest percentage at 36.6% followed by vitamins and minerals at 16.4%, herbal drugs at 14.0% and haematinics at 13.8%. The classes of medicines held least at home were antispasmodics and anti-asthmatics at 0.2% each, and antidiabetics and antacids at 0.3% and 0.4%, respectively. From Table 3 Panel B, the majority of medicines, 73.0%, were obtained from patent proprietary medicine vendors (PPMVs) while only 10.5% were obtained from a health facility with 4.0% from a public facility and 6.5% from a private facility. The majority of medicines obtained through other means were prepared at home, obtained from a family member, or from a private medical professional.

**Table 3:** Characteristics of all drug responses.

**Panel A. Drug class**	**Total N (%)**^**a**^	**Correct Match N (%)**	**Incorrect Match N (%)**	**Indeterminate N (%)**	**P-value**
Analgesic	3,393 (36.6)	3,226 (95.1)	53 (1.6)	114 (3.4)	< .0001
Antacid	35 (0.4)	25 (71.4)	6 (17.1)	4 (11.4)	
Anti-asthmatic	15 (0.2)	13 (86.7)	1 (6.7)	1 (6.7)	
Anti-diabetic	31 (0.3)	23 (74.2)	3 (9.7)	5 (16.1)	
Antihistamines	120 (1.3)	28 (23.3)	43 (35.8)	49 (40.8)	
Anti-hypertensive	334 (3.6)	293 (87.7)	25 (7.5)	16 (4.8)	
Anti-malarial	462 (5.0)	444 (96.1)	15 (3.3)	3 (0.7)	
Anti-spasmodic	19 (0.2)	14 (73.7)	4 (21.1)	1 (5.3)	
Antibiotics	692 (7.5)	443 (64.0)	184 (26.6)	65 (9.4)	
Expectorant	72 (0.8)	47 (65.3)	23 (31.9)	2 (2.8)	
Haematinics	1,276 (13.8)	651 (51.0)	237 (18.6)	388 (30.4)	
Herbal	1,297 (14.0)	0 (0)	0 (0)	1,297 (100.0)	
Vitamins and minerals	1,520 (16.4)	460 (30.3)	511 (33.6)	549 (36.1)	
Total	9,266 (100.0)	5,667 (61.2)	1,105 (11.9)	2,494 (26.9)	
**Panel B. Place where drug was obtained**					
Public clinic	366 (4.0)	242 (66.1)	58 (15.9)	66 (18.0)	< .0001
Private clinic	597 (6.5)	409 (68.5)	80 (13.4)	108 (18.1)	
Patent Proprietary Medicine Vendors (PPMVs)	6,695 (73.0)	4,796 (71.6)	892 (13.3)	1,007 (15.0)	
Other	1,514 (16.5)	155 (10.2)	58 (3.8)	1,301 (85.9)	
Total	9,172 (100.0)	5,602 (61.1)	1,088 (11.9)	2,482 (27.1)	
Missing	94	65	17	12	

^a^Value represents percentage of the total number of drugs.

As shown in Columns (2–5) of Table 3, 61.2% of the medicines observed had a correct match, 11.9% had an incorrect match and 26.9% could not be determined. Antimalarial drugs were most likely to have a correct match at 96.1% followed by analgesics at 95.1%, antihypertensives at 87.7% and anti-asthmatics at 86.7%. Conversely, antihistamines were most likely to have an incorrect match at 35.8%, followed by vitamins and minerals at 33.6%, expectorants at 31.9% and antibiotics at 26.6%. All the herbal drugs could not be determined. Medicines obtained from a PPMV were most likely to correctly match at 71.6%, followed by those obtained from a private facility at 68.5%, public facility at 66.1% and those obtained through other means at 10.2%.

### Factors that influence the likelihood of having a medicine at home

Table 4 shows three mixed-effects logit models for the likelihood of having a medicine at home. In Model (1), we controlled for only demographic predictors, in Model (2) for all demographic, socioeconomic, health-related and treatment area predictors and in Model (3), for health insurance status in addition to Model (2) predictors. We also show, only in cases of a significant result, the interaction of the predictor variables with living in the treatment area and being enrolled in health insurance.

**Table 4:** Modelling the likelihood of having a medicine at home at the individual level.

	Likelihood of having a medicine at home (Odds Ratio)		
Predictor	Model 1	Model 2	Model 3
Demographic Characteristics			
**Age Group**			
0–18	-	-	-
19–35	1.17 [0.96–1.42]	1.10 [0.84–1.43]	1.10 [0.86–1.40]
36–60	2.03*** [1.72–2.40]	1.58** [1.16–2.15]	1.60** [1.17–2.18]
>60	2.51*** [1.95–3.24]	2.02*** [1.41–2.91]	2.04*** [1.42–2.93]
**Gender**			
Male	-	-	-
Female	1.09 [0.96–1.24]	1.04 [0.90–1.19]	1.04 [0.90–1.19]
Socioeconomic Characteristics			
**Marital Status**			
Single		-	-
Married		1.23 [0.95–1.60]	1.22 [0.94–1.58]
**Household Size**			
1–5		-	-
>5		0.77* [0.59–0.99]	0.77 [0.59–1.00]
**Education Level**			
No education		-	-
Primary education		0.82* [0.68–0.99]	0.83* [0.69–0.99]
Secondary education		0.65*** [0.51–0.82]	0.66*** [0.52–0.84]
Tertiary education		0.86 [0.53–1.39]	0.86 [0.53–1.38]
**Income Level**			
Quintile 1 (Poorest)		-	-
Quintile 2		1.39 [0.86–2.24]	1.37 [0.85–2.22]
Quintile 3		1.42 [0.89–2.27]	1.38 [0.86–2.21]
Quintile 4		1.41 [0.88–2.24]	1.36 [0.86–2.18]
Quintile 4##living in treatment area		0.30* [0.10–0.90]	
Quintile 5 (Richest)		2.13*** [1.33–3.39]	2.05*** [1.28–3.28]
Health-related Characteristics			
**Chronic Disease**			
No		-	-
At least one		1.79*** [1.30–2.46]	1.75*** [1.27–2.41]
Have chronic disease##insured			2.51* [1.11–5.66]
**Distance to Nearest Health Facility** (Km)		0.98 [0.95–1.02]	0.98 [0.95–1.02]
Insurance program indicators			
**Individual in Treatment Area**			
No		-	-
Yes		0.33*** [0.24–0.44]	0.31*** [0.23–0.42]
**Individual has Health Insurance**			
No			-
Yes			1.24 [0.94–1.63]
Chi-square	105	185	190
df	4	16	17
Deviance	5,356	4,888	4,882
N	4,641	4,512	4,510

* p<0.05

** p<0.01

*** p<0.001 [95% confidence interval].

Results of fitting a series of logistic regression models predicting the likelihood of having a medicine at home and controlling for relevant covariates. Model 1: Only demographic, Model 2: All socio-demographic, Model 3: Socio-demographic and insurance status.

Results from Models (1) to (3) showed that age was a consistently positive predictor of having a medicine at home, with the highest odds for those older than 60 years (odds ratio: 2.04, CI: 1.42–2.93) followed by individuals in the 36 to 60 years age group (odds ratio: 1.60, CI: 1.17–2.18) when compared to those aged 0 to 18 in the full Model (3). Gender was not associated with having a medicine at home.

At the socioeconomic level, education and income were consistent predictors of having a medicine at home. The likelihood of having a medicine decreased as the education level increased except for individuals with tertiary education. From Model (3), individuals with primary education had an odds ratio of 0.83 (CI: 0.69–0.99) while those with secondary education had lower odds ratio of 0.66 (CI: 0.52–0.84) relative to individuals with no completed education. The likelihood of having a medicine at home also increased with income level but was only significant for those in the highest income level (odds ratio: 2.05, CI: 1.28–3.28) compared to those in the lowest income level.

Using the full Model (3) also for the health-related covariates, individuals with one or more chronic diseases had a higher likelihood of having a medicine at home (odds ratio: 1.75, CI: 1.27–2.41) relative to those without chronic disease. Distance to the nearest health facility was not associated with the likelihood of having medicines at home. Living in a treatment area–i.e. benefiting from the health facility quality upgrade of the insurance program without necessarily being insured oneself–was negatively associated with having medicines at home (odds ratio: 0.31, CI: 0.23–0.42), while having insurance was not a significant predictor.

In the interaction models, there was no statistically significant heterogeneity across any of the predictor variables, except for individuals in the treatment area who belonged to the fourth highest income quintile (odds ratio: 0.30, CI: 0.10–0.90). In addition, interacting chronic disease status with having health insurance, showed that the likelihood of having a medicine at home was especially pronounced for individuals who had a chronic illness and who were enrolled in health insurance (odds ratio: 2.51, CI: 1.11–5.66).

### Determinants of correct drug match

Table 5 shows logit regressions of the probability that a medicine’s use correctly matched its clinical purpose on an increasing number of explanatory variables: drug class in Model (1), drug class plus demographic, socioeconomic, health-related and treatment area predictors in Model (2), and all predictors from Model 2 plus health insurance status indicator in Model (3). As part of Models (2) and (3), we also present, only in cases of a significant result, the interaction of the predictor variables with living in the treatment area and being enrolled in health insurance, respectively.

**Table 5:** Modelling the determinants of correct drug match at the medicine level.

Predictor	Model 1	Model 2	Model 3
Drug Characteristics			
**Drug Class**			
Analgesic	-	-	-
Antacid	0.07*** [0.03–0.18]	0.07*** [0.02–0.17]	0.07*** [0.03–0.18]
Anti-Asthmatic	0.21 [0.03–1.70]	0.22 [0.03–1.83]	0.22 [0.03–1.87]
Anti-Diabetic	0.13** [0.03–0.46]	0.12** [0.03–0.46]	0.12** [0.03–0.45]
Antihistamine	0.01*** [0.01–0.02]	0.01*** [0.01–0.02]	0.01*** [0.01–0.02]
Anti-Hypertensive	0.19*** [0.10–0.37]	0.23*** [0.10–0.54]	0.23*** [0.10–0.54]
Anti-Hypertensive##Treatment		9.95** [1.88–52.69]	
Anti-Hypertensive##Being insured			25.15*** [4.17–151.61]
Anti-Malarial	0.49* [0.27–0.89]	0.44* [0.24–0.82]	0.44* [0.24–0.83]
Anti-Spasmodic	0.06*** [0.02–0.19]	0.08*** [0.02–0.32]	0.08*** [0.02–0.33]
Antibiotic	0.04*** [0.03–0.06]	0.04*** [0.03–0.06]	0.04*** [0.03–0.06]
Antibiotic##Being insured			3.60** [1.45–8.92]
Expectorant	0.03*** [0.02–0.07]	0.03*** [0.01–0.06]	0.03*** [0.01–0.06]
Haematinic	0.05*** [0.03–0.07]	0.04*** [0.03–0.07]	0.04*** [0.03–0.07]
Haematinic##Treatment		2.32* [1.03–5.25]	
Haematinic##Being insured			5.40*** [2.54–11.50]
Vitamin & Mineral	0.02*** [0.01–0.02]	0.01*** [0.01–0.02]	0.01*** [0.01–0.02]
Vitamin & Mineral##Being insured			3.76*** [1.88–7.53]
**Place Drug was Obtained**			
Public health facility		-	-
Private health facility		2.01* [1.08–3.77]	2.04* [1.10–3.80]
Patent Proprietary Medicine Vendors		1.74* [1.05–2.90]	1.94* [1.15–3.25]
Other		1.37 [0.73–2.58]	1.51 [0.80–2.85]
Demographic characteristics			
**Age Group**			
0–18		-	-
19–35		1.07 [0.71–1.61]	1.07 [0.71–1.62]
36–60		0.76 [0.51–1.13]	0.77 [0.52–1.15]
≥61		0.70 [0.47–1.04]	0.70 [0.47–1.04]
**Gender**			
Male		-	-
Female		0.95 [0.77–1.16]	0.94 [0.77–1.15]
Socioeconomic characteristics			
**Marital Status**			
Single		-	-
Married		0.97 [0.70–1.35]	0.97 [0.70–1.34]
**Household Size**			
1–5		-	-
≥6		0.97 [0.73–1.29]	0.98 [0.73–1.30]
**Education Level**			
No education		-	-
Primary		1.01 [0.80–1.28]	1.01 [0.79–1.28]
Secondary		0.85 [0.63–1.13]	0.86 [0.64–1.15]
Tertiary		1.63* [1.06–2.49]	1.61* [1.05–2.47]
**Income Level**			
Quintile 1 (Poorest)		-	-
Quintile 2		1.13 [0.69–1.87]	1.13 [0.68–1.86]
Quintile 3		1.14 [0.72–1.80]	1.13 [0.72–1.79]
Quintile 4		1.38 [0.88–2.18]	1.37 [0.87–2.15]
Quintile 5 (Richest)		1.34 [0.85–2.09]	1.31 [0.84–2.06]
Health-related characteristics			
**Chronic Disease**			
No		-	-
At least one		0.95 [0.71–1.27]	0.93 [0.70–1.25]
**Distance from Nearest Health Facility (Km)**		0.98 [0.95–1.02]	0.98 [0.95–1.02]
Insurance program indicators			
**Observation in Treatment Area**			
No		-	-
Yes		1.51** [1.16–1.98]	1.41* [1.05–1.87]
**Insurance Status**			
No			-
Yes			1.26 [0.93–1.71]
Chi-square	835	892	892
df	11	30	31
Deviance	4,242	3,998	3,990
N	6,772	6,524	6,517

* p<0.05

** p<0.01

*** p<0.001 [95% Confidence Interval].

Results of fitting a series of logistic regression models predicting the likelihood of having a medicine use correctly match its actual clinical use, using drug class, and controlling for relevant covariates. Model 1: Drug class only, Model 2: Drug class and socio-demographic factors, Model 3: Drug class, socio-demographic and insurance status.

Drug class was strongly associated with the likelihood that a medicine had a correct drug match. Compared to analgesics, the most commonly found medicine at home, all other drug classes had lower odds of a correct match: e.g. the full Model (3) finds an anti-malarial odds ratio = 0.44 (CI: 0.24–0.83), antihypertensives odds ratio = 0.23 (CI: 0.10–0.54), and antibiotics odds ratio = 0.04 (CI: 0.03–0.06) relative to analgesics.

Public facilities were taken as the reference category for the place where the medicine was obtained. From Model (3), compared to public facilities, the highest likelihood of a correct match was found for medicines obtained from private clinics (odds ratio: 2.04, CI: 1.10–3.80) followed by those obtained from PPMVs (odds ratio: 1.94, CI: 1.15–3.25); there was no significant association for those obtained through other means.

There was no significant association of correct drug match with age, gender, marital status, household size, income level or chronic illness. From Model (3), correct drug match was however most likely for medicines found with individuals with tertiary or higher education (odds ratio: 1.61, CI: 1.05–2.47) relative to those with no education.

Living in a treatment area was positively associated with correct drug match (odds ratio: 1.41, CI: 1.05–1.87), while being insured did not have a significant relationship with correct drug match on average. However, these average effects mask some heterogeneity. From interaction Model (2), medicines such as antihypertensives (odds ratio: 9.95, CI: 1.88–52.69) and haematinics (odds ratio: 2.32, CI: 1.03–5.25) had a higher likelihood of being correctly matched when found with individuals living in the treatment area compared to the control area. Also, being insured had a strong positive effect on specific drug classes as shown in Model (3): Antihypertensives (odds ratio: 25.15, CI: 4.17–151.61), antibiotics (odds ratio: 3.60, CI: 1.45–8.92), haematinics (odds ratio: 5.40, CI: 2.54–11.50), and vitamins and minerals (odds ratio: 3.76, CI: 1.88–7.53) had a significantly higher likelihood of a correct match when found with insured individuals compared to uninsured individuals.

## Discussion

This paper described a unique study performed in rural Nigeria aimed at understanding the type of medicines that people had at home, the factors that influence the likelihood of having medicines at home, the extent to which the self-reported use of the medicines correctly matched their clinical purpose and the factors that determine this match. It also examined whether the likelihood of having medicines at home or having a correct drug match was affected by a health insurance program that offered quality upgrades for selected health facilities in program areas as well as financial protection for individuals through subsidized health insurance.

The results showed that the majority of the medicines found at home comprise analgesics, vitamins and minerals, haematinics, and herbal drugs. Most of these medicines were obtained from PPMVs in the communities. These findings align with those from other studies carried out within and outside Nigeria [22, 23]. Our results also showed that about 61% of the medicines held in the homes of the respondents had a correct match while the reported use of about 12% of the medicines held at home did not match their clinical use. The match of the remaining 27% of medicines was indeterminate. Having medicines used not according to the appropriate clinical purpose can increase the risk of irrational drug use in local communities [1, 16, 17].

In addition, there exists a clear divide between medicines that were more likely to correctly match and those less likely to correctly match. While analgesics, anti-malarial, antihypertensives, anti-asthmatic and antidiabetics, were relatively likely to be used according to their clinical purpose, vitamins and minerals, antispasmodics, antihistamines, expectorants, and antibiotics were less likely to be used according to their clinical purpose. The medicines in the former category included medicines that were used to treat specialized conditions like hypertension. People who had such conditions might be more likely to know how and when to use their medications.

One of the medicine classes with a lower likelihood of correct use was antibiotics. This is especially worrisome in view of the global increase in antimicrobial resistance (AMR) [37]. AMR has been associated with high risk of mortality and increased economic costs [38]. To reduce the prevalence of inappropriate antibiotic use or medicine use in general, there is hence an urgent need for policymakers and health providers to enhance health literacy and provide information to individuals on different medicine types and their proper use.

As a crucial feature of our study, the dataset we used was collected for the purpose of an impact evaluation of a comprehensive subsidized voluntary health insurance program implemented in Kwara State since 2009. Our findings underscore the importance of having upgraded health facilities and providing financial protection through health insurance to community members. We found that people living in treatment areas where facilities were upgraded were less likely to have medicines at home compared to individuals in comparison areas. However, having health insurance increased the likelihood of having medicines at home for the chronically ill. This suggests that better quality formal care is associated with lower amounts of medicines kept in the home, while improved financial access due to health insurance increases medicines at home for the chronically ill. Indeed, a study on access to chronic medicines in five developing countries found the likelihood of having chronic medicines at home to be higher for people with health insurance coverage [39].

Moreover, we found that the health insurance intervention, by providing facilities with a quality upgrade or risk coverage of individuals, resulted in a higher likelihood that specific medicines were used according to clinical purpose. By upgrading the facilities, this effect was strongly significant for antihypertensives–one of the core focal points of the program [33], while by improving risk coverage, the effect was very pronounced for antihypertensives, antibiotics, haematinics, and vitamins and minerals. To our knowledge, this is the first study that investigated the role of a combined insurance and quality upgrade program on correct drug use. Upgrading a health facility means having the right infrastructure and adequate number of skilled health workers motivated to provide support to patients on their care and the use of medicines [40]. Health insurance can help to improve access to medicines for the chronically ill and when implemented in areas with quality health facilities, it may serve as a means to reduce irrational drug use. As such, investing in comprehensive insurance schemes may be an important strategy in the fight against AMR.

Delving deeper into the determinants of medicine use, we observed a positive relationship between age and the likelihood of having medicines at home but no significant relationship with having a correct match. This is probably because older people are at a higher risk of becoming chronically ill and as a result, would require more care and more medications for use [41]. Indeed, a related study using the 2009 data from the same study area found that the prevalence of chronic conditions increased systematically from less than 2 percent among individuals below 20 years old to 27 percent among the 70-year old and above [42].

In terms of gender, we found no significant relationship with either having medicines at home or having a correct match. This finding adds to the inconclusive findings from other studies that found that the misuse of medicines was most associated with either the male or instead the female gender [22, 25].

With regards to education, we found that those with no education were more likely to have medications at home, and less likely to correctly use medicines in line with their clinical purpose compared to those with higher levels of education. Indeed, lack of education has been linked with lower health literacy and the inability to understand health information [43]. As a result, people with limited education may have a poorer understanding of their health and may find it difficult to read and understand the labels on a medication. Without proper education or guidance from a medical professional, this could lead to a higher likelihood of irrational drug use.

We found a positive relationship between income and having medications at home but no significant relationship with having a correct match. People at the higher end of the wealth distribution probably have more medications at home because of their earning power and higher level of disposable income that can be used to buy medications out-of-pocket without worrying about catastrophic health expenditure. This finding is in line with other studies that showed that richer households were more likely to have medicines stored at home [44]. Conditional on income, we observed that individuals in larger-sized households were less likely to have medicines at home. This might capture differential effects of household composition where larger household sizes are often correlated with a higher number of dependents. This in turn could reflect a lower ability to afford buying medicines, particularly if there is only one breadwinner in the family.

We observe that distance to a nearest health facility had no significant relationship with having medicines at home. This could be because many people get their medicines from PPMVs rather than from other channels like the health facility. Indeed, several studies have documented the preponderance of PPMVs in communities and their role in access to medicines [8, 10].

With regards to the place where medicines were obtained, it is a concern that incorrectly matched drugs were mostly gotten at public facilities. This may suggest that either incorrect medicines were prescribed to individuals at the public facility level or that the medicines provided were not well explained to the patient on what they should be used for. This corroborates findings from several studies that have documented prescribing patterns of medical doctors in Nigeria, which deviate from WHO’s recommended practices [45–47].

There are several limitations to this study. First, this study focuses on a narrow definition of drug misuse, which links actual use to clinical purpose. As a result, even though we were able to determine correct drug match among the medicines that the respondents had in their homes, we could not determine whether the medicines were being overused, underused or even used based on a medical professional’s recommendation. In addition, we did not look at the quality of the medicines in terms of expiry date and conditions of storage. Another limitation is the self-reported nature of actual use, which might be affected by respondents’ ability to accurately recall the purpose for which they were using the medicines. Especially when recall periods lengthen, accuracy might go down [48]. In addition, most patients report symptoms and not diseases, so it is possible that the chronically ill patients may have inaccurately reported the type of illness that they had. Thirdly, in comparing the treatment and control areas, although very similar, we cannot exclude that there may have been differential changes over time. Nevertheless, our results are in line with other impact studies based on the same data that find positive effects on access to formal health care and improved financial affordability of care [27]. Lastly, having health insurance was a voluntary decision, which means that there may have been selection bias as the very elderly and sick patients who were more likely to need medicines may have been more incentivized to enroll in the scheme. As a result, there should be caution in the interpretation of our results as our findings suggest associations rather than causal effects. Despite these limitations, we believe that our findings are useful for governments and policymakers in understanding the menace and likely interventions for addressing irrational drug use.

## Conclusion

This study provides insights into the medicines that are commonly held at home, their likelihood to be used correctly and the impact of a health insurance program on correct use among households in rural North-Central Nigeria. To our knowledge, this is the first study that investigated the role of a coordinated health insurance program/provider upgrading effort on correct match of medicines kept at home. From our results, analgesics are the most common medicine class at home while anti-malarial are most likely to have a correct match, especially in areas where the program has upgraded the selected health facilities. Medicines like antibiotics are likely to have an incorrect match and as such pose a threat to the public health system because of the increasing risk of antimicrobial resistance.

To address the menace of irrational drug use, we recommend that policy leaders work to educate and improve the health literacy of local community members. Since PPMVs are prominent in the communities, we also recommend that policy makers develop effective strategies to contract and work with these vendors to educate community members on different medicine types and their use. This could for example take the form of a combination of training and licensing for PPMVs, or a hub and spokes model in which insurance schemes reach beyond selected health facilities to also cover certified PPMVs. Finally, as governments move towards achieving universal health coverage, we recommend the design and implementation of health insurance programs that both enhance risk-pooling for increased financial protection and improve access to better-quality health care from upgraded providers. Doing this may provide a unique opportunity to reduce the burden of incorrect drug use.

## Supporting information

S1 File Minimal dataset for reproduction of results. Dataset containing variables used for analysis in Excel format.
(XLSX)

## List of abbreviations

AMR: Antimicrobial Resistance
EA: Enumeration Areas
HIF: Health Insurance Fund
KSHIP: Kwara State Health Insurance Program
PPMV: Proprietary Patent Medicine Vendor
SD: Standard Deviation
UHC: Universal Health Coverage
